# Do coxibs reduce prescription of gastroprotective agents? Results of a record linkage study

**DOI:** 10.1186/1478-7547-4-4

**Published:** 2006-03-22

**Authors:** Giulio Formoso, Angelo Menna, Claudio Voci, Andrea Violante, Nicola Magrini

**Affiliations:** 1 CeVEAS (Centro per la Valutazione della Efficacia della Assistenza Sanitaria), Modena, Italy

## Abstract

**Background:**

Coxibs are claimed to be cost-effective drugs and reduced prescription of gastroprotective agents is assumed to be one of their major benefits. Real life prescription of these drugs may be substantially different than that considered in pharmacoeconomic analyses or claimed by drug companies, yet. Our objective was to evaluate whether coxibs were associated with reduced prescription of gastro-protective agents (GPAs, specifically proton pump inhibitors, H_2 _blockers and misoprostol) compared to non selective NSAIDs.

**Methods:**

A record-linkage study was performed using 2001 outpatient prescription data from the province of Modena (about 632,000 inhabitants, in Northern Italy). Logistic regression was used to calculate the odds ratio of GPA prescription for coxib and non-selective NSAID adult users (> 14 years). Three categories of users were further investigated: "acute", "chronic and "incident or new". Main outcome measures were same-day co-prescription and 30 days prescription of GPAs in coxibs and non selective NSAIDs users. To limit selection bias, data were adjusted for age, sex, DDD of coxibs and non selective NSAIDs received during 2001, DDD of GPAs and (for non-incident users) DDD of NSAIDs received during the previous 4 years

**Results:**

Same day co-prescription rates were similar considering the overall population and "acute" users. Chronic coxibs users instead showed higher co-prescription rates than chronic NSAIDs users (OR = 1.2, p < 0.05). GPA prescription within thirty days was also higher among all subgroups of coxibs users (OR ranging from 1.6 to 2.0, p < 0.001).

**Conclusion:**

Assumptions made in pharmacoeconomic analyses on coxibs (lower GPA prescription associated with coxibs use) may be overly optimistic. Claims made through cost-effectiveness data should be carefully interpreted, and mechanisms for attributing drug prices revised accordingly.

## Background and objective

Pharmacoeconomic analyses are used to highlight potential advantages of new drugs over older ones by showing, in general, that higher effectiveness and/or less frequent side effects may be worth the (generally) higher cost. Assumptions and findings from these analyses may not reflect everyday practice yet, as real life prescription and use of marketed drugs may be different from that observed in Randomised Controlled Trials and/or assumed in economic models.

Coxibs were found to have higher gastrointestinal tolerability than traditional NSAIDs, but their overall safety profile is controversial in light of cardiovascular risks demonstrated for rofecoxib, celecoxib and (in post surgical patients) for parecoxib and valdecoxib [1-5]. These are claimed to be cost-effective drugs especially in high risk patients, and especially on the ground of reduced co-prescription of gastro-protective agents (GPAs), as some cost-effectiveness analyses stated [6-9] and pharmaceutical companies proposed [10] (especially considering patients at high risk of gastrointestinal bleeding). NSAIDs prescription and co-prescription of GPAs are relevant to decision makers: GPAs and anti-inflammatory drugs prescribed within the National Health System (NHS) account for 7.1% and 4.5% of the Italian gross pharmaceutical expenditure, respectively [11].

In Italy, the co-prescription of GPAs and coxibs is theoretically not allowed, since GPAs prescription should be justified – on the prescription itself – on clinical grounds other than the use of a coxib. Often in practice, doctors do not follow this rule and co-prescribe GPAs with coxibs. Our purpose was to explore whether coxibs are associated with (at least)reduced co-prescription rates of GPAs in comparison with traditional NSAIDs, thus testing one of the main assumptions of pharmacoeconomic analyses on these drugs.

## Methods

We performed a record-linkage study using 2001 NHS prescription data from an electronic database of outpatient prescriptions of the province of Modena (about 632,000 inhabitants, in Northern Italy). Specifically, we analysed prescriptions of GPAs (proton pump inhibitors, H_2 _blockers and misoprostol), occurring either the same day of, or within 30 days since (assumed as an adequate time window for acute gastrotoxic events), prescription of oral NSAIDs and/or coxibs. Information about time and amount of prescribing, and age and sex of recipients were collected. All these prescriptions are free of charge within our Regional Health System.

Logistic regression was used to calculate the odds ratio of GPA prescription for coxib and non-selective NSAID users, excluding the pediatric population (< 14 years old). Specific subgroups were investigated: "acute" users, defined as those who received less than 60 Defined Daily Doses (DDD) of traditional NSAIDs or coxibs during 2001; "chronic" users, as those who received at least 60 DDD of any anti-inflammatory drug during 2001 (96% of them had received at least a two-pieces repeated prescription over four months); "incident"/new users, as those who had never been prescribed any NSAIDs during the previous four years; in this latter group, we further distinguished those who had never been prescribed GPAs in the same period (theoretically, at lower risk of assuming GPAs in the observational period). In addition to considering those subgroups, further attention was given to limit selection bias (prescription of coxibs to patients at higher risk of GI bleeding) by adjusting for age, sex, DDD of coxibs and non selective NSAIDs received during 2001, DDD of GPAs and [*] (for non-incident users) DDD of NSAIDs received during the previous 4 years.

## Results

In 2001, coxibs and oral non-selective NSAIDs were prescribed to 3.4% and 9.2% of the adult population of the study area, respectively. The vast majority of patients treated with these drugs (73.5%) were acute users (less than 60 DDD/year, with an average of 26 DDD); almost no difference existed in the proportion of coxibs and non-selective NSAIDs acute users. About 50% of coxibs and NSAIDs users were "incident" users (subjects having not received any NSAIDs in the previous four years).

GPAs were co-prescribed to 6.1% of individuals receiving anti-inflammatory drugs, and 5.7% were prescribed GPAs within 30 days (Table 1). The majority (73.3%) of gastro-protected patients received proton pump inhibitors, whereas 26.8% and 5.2% received H_2 _receptor antagonists and misoprostol, respectively (some patients have taken different classes of GPAs at different times). Table 2 shows adjusted odds ratio of GPA prescription for coxib and non selective NSAIDs users. Co-prescription of GPAs was similar for coxib and non-selective NSAID users in all patients subgroups, except in incident users (higher GPAs prescription in coxib users; OR = 1.2, p = 0.03). Conversely, prescription of GPAs within 30 days was always higher – across all patients' subgroups – for coxib compared to non-selective NSAID users (ORs range from 1.6 to 2.0).

**Table 1:** Prescription of GPAs (%) according to type of NSAIDs used

User category	N. users	Same day GPA co-prescription	Prescription of GPA within 30 gg
Only traditional NSAIDs	50,204	2400 (4.8%)	2018 (4.0%)
Only coxibs	13,339	829 (6.2%)	888 (6.7%)
Both traditional NSAIDs and coxibs	8,058	1153 (14.3%)	1172 (14.5%)
Total	71,601	4382 (6.1%)	4078 (5.7%)

**Table 2:** Odds ratio (95% CI) of GPA prescription (proton pump inhibitors, H_2 _blockers and misoprostol) – occurring the same day or within 30 days – for coxib vs. oral non selective NSAIDs adult users

			Adjusted ^# ^odds ratio (95% CI) of GPA prescription (coxib vs. non selective NSAIDs users)	
*Patients' profile*	*N*.	*%*	*Co-prescription of GPAs (same day)*	*Prescription of GPAs within 30 days*
			
No NSAIDs and/or GPAs in the previous 4 years	30,658	42.8	1.1 (0.9–1.3)	1.7 (1.3–2.1) °
No NSAIDs in the previous 4 years	36,077	50.4	1.2 (1.0–1.4) *	1.6 (1.3–1.8) °
Acute patients †	52,608	73.5	1.3 (1.0–1.7)	1.7 (1.3–2.3) °
Chronic patients ‡	18,993	26.5	1.2 (1.0–1.5) *	1.7 (1.3–2.3) °
All patients	71,601	100	1.1 (1.0–1.2)	2.0 (1.8–2.2) °

† Individuals who had been prescribed less than 60 DDD of NSAIDs (either selective or non selective) during 2001

‡ Individuals who had been prescribed at least 60 DDD of NSAIDs (either selective or non selective) during 2001

# Adjusted for age, sex, DDD of coxib and NSAID received during 2001, and (not for incident patients) DDD of GPAs and NSAIDs received during the previous 4 years

* p < 0.05

° p < 0.001

## Discussion

Even with the intrinsic limitations of such a record-linkage study (residual confounding; analysing only prescriptions and not diagnoses), our example suggests that coxibs may not be associated with reduced prescription of GPAs – as manufacturers claim or imply – and that preventive (and selective) co-prescription of GPAs and coxibs to those at higher risk of bleeding is not necessarily the key to the whole story. Unexpectedly, patients who received coxibs (supposed to be less gastrotoxic than traditional NSAIDs) were more "at risk" to be prescribed GPAs within 30 days. Moreover incident NSAIDs users, supposed to be less at risk of being prescribed GPAs (thus being less affected by selection bias), were actually co-prescribed GPAs more often the same day. This could mean that they are either not safer than traditional NSAIDs (in terms of gastrointestinal tolerability) or that anyway prescribers do not reduce co-prescription of GPA. Although gastrointestinal tolerability of coxibs is controversial, both RCT and observational studies exclude that coxibs are more gastrotoxic [12]. On the other side, evidence of higher gastrointestinal tolerability from RCTs is only available for rofecoxib, but this drug has been withdrawn from the market worldwide on September 2004 for evidence of increasing cardiovascular risk.

Is it true that more expensive drugs eventually lower prescription costs? Such an assumption is often made in pharmacoeconomic analyses and has also been made in economic analysis on coxibs. The purpose of our study was to explore this assumption and our data, consistent with those of previous researches [12,13] (but adding further insight on drug prescriptions in acute, chronic and incident users) show it may not be true in practice. Attitudes towards "defensive" medicine could be a key determinant, so that some doctors may consider this prescribing behaviour safer, also considering the high tolerability of proton pump inhibitors. This may indirectly result into prescribing coxibs *plus *GPAs, even if in Italy this represents an unlicensed use, and despite lacking evidence from RCTs on the higher safety profile of this combination compared to coxib alone or to traditional NSAIDs plus GPAs. In this regard, no evidence-based guideline recommends such a combination as a therapeutic alternative to NSAIDs/GPA combination. In actual practice, doctors may also be influenced by patients and especially drug representatives [14,15] in using the (supposedly) best and more expensive (but without a solid base of evidence) drugs.

## Conclusion

We think more research and debate should be made on methods used in pharmacoeconomics – which may be used to affect decisions on drug reimbursement and prices [16] – and on how to best integrate effectiveness and economics data. We suggest that observational data should be evaluated while developing a pharmacoeconomic model if resource consumption is at stake, to get a more realistic "flavour" of drug utilization in clinical practice and related economic implications. When RCT data on resource consumption are available, these should at least be compared to observational data – evaluating their overall consistency – before incorporating them in economic models. Pharmacoeconomic analyses may then lose some of their appeal, especially for manufacturers, [17] but they would probably be more useful in providing decision makers with reliable data.

## Conflict of interest statement

All of the authors work within a NHS research centre. None of them had any financial and personal relationships with other people or organisations that could inappropriately influence (bias) their work.

## Authors' contributions

**GF **coordinated study design, coordinated and did the statistical analysis, wrote the initial draft of the paper and revised the final version.

**AM **participated in study design, extracted the data, coordinated and did the statistical analysis

**CV **participated in study design, extracted the data and did the statistical analysis

**AV **extracted the data, did the statistical analysis and revised the final version

**NM **had the original idea, coordinated study design and revised the final version;

The article was read and approved by all contributors.
